# Hydrometallurgical Strategy To Reduce Waste through the Recycling of Lithium Iron Phosphate Batteries

**DOI:** 10.1021/acsomega.5c07786

**Published:** 2025-12-23

**Authors:** David da Silva Vasconcelos, Denise Crocce Romano Espinosa, Jorge Alberto Soares Tenório, Amilton Barbosa Botelho Junior, Luciana Assis Gobo

**Affiliations:** 1 Chemical Engineering Department, Polytechnique School, University of São Paulo, Reitoria Street, 374, Butantã, São Paulo 05508-220, Brazil; 2 Department of Chemical Engineering, Norwegian University of Science and Technology, Trondheim 7491, Norway; 3 TUPY S/A, Rua Albano Schmidt, 3400, Joinville, Santa Catarina 89227-901, Brazil

## Abstract

Batteries with LiFePO_4_ as active material stand out due to the absence of critical materials, such as nickel and cobalt, thermal stability, and security. In the next years, high volumes of LFP batteries will reach their end of life, and overall material recovery will contribute to meeting the Li demand and reducing the CO_2_ footprint. Recovery of 97% of plastics and 85.3% graphite prevented materials from burning in furnaces and reduced the CO_2_ footprint from recycling. Leaching cathode active material using H_2_SO_4_ without H_2_O_2_ resulted in active material leaching with reduced metallic foil solubilization and less reagent consumption. Redirecting H_2_O_2_ consumption to Fe removal by precipitation, combined with ion exchange columns at 25 °C, successfully deepened Fe purification from solution. Precipitation of Al recovered 15.3% as an Al­(OH)_3_ coproduct. After evaporation in a real solution, 72.2% of Li was precipitated as Li_2_CO_3_, contributing to increasing the recycling share in the Li supply.

## Graphical abstract

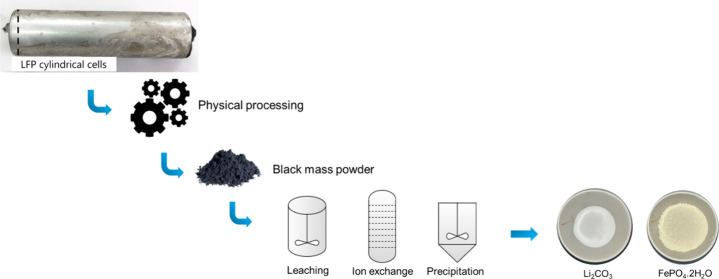

## 1 Introduction

Renewable power sources such as wind and solar, integrated with the transport sector to recharge electric vehicles (EVs) and eliminate the CO_2_ pipe emissions, represent a significant step toward the sustainability of humans on Earth. This transition started a few years ago, and nowadays, the transport sector is under electrification with over 26 million EVs on the roads and sales reaching 14% of the global car market share in 2022.^1^

Lithium-ion batteries (LIBs) dominate the EV market due to their high energy density, low self-discharge, safe handling, and longer life cycles compared to lead acid, nickel–metal hydride, and cadmium batteries. Despite that, transition metals in the cathode make the fabrication of new LIBs dependent on mineral extraction, stimulating countries to classify them as critical raw materials (strategic materials or undersupply risk in the short and medium term).^2^

The efforts to reduce cobalt (Co) content in LIBs made cathode active materials with high nickel (Ni) content, such as nickel manganese cobalt oxide with high Ni content (NMC 811), lithium nickel cobalt aluminum oxide (NCA), and lithium iron phosphate (LFP), more attractive for vehicle application.^1^ Beyond the market movement, Chinese companies BYD and CATL reduced Co and Ni dependence by investing in LFP packs with higher performance, cell-to-pack assembled in vehicles with no modules to improve energy density.^1^ Li is essential for the LIB chain, and the demand is expected to be 55% higher than production by 2030. In this scenario, recycling of spent batteries is a solution to recover Li toward circular economy.

Current industrial recycling processes are pyrometallurgical.^3^ The main disadvantages include Li losses on the slag phase, CO_2_ footprint from furnace heat, graphite (C) and plastic burning, and toxic gas release from electrolyte decomposition.^4^ An alternative way is hydrometallurgical recycling, which has less energy consumption, a lower carbon footprint, and the possibility of lithium (Li) recovery,^5^ producing single metal salts (FePO_4_, Li_2_CO_3_, Al­(OH)_3_), which are more valuable than metallic alloys.^6^ In addition, graphite (C) and plastic recovery contribute to the reduction of the estimated 6.5 GtCO_2_ from the plastic lifecycle by 2050 ^7^ and release from the graphitization process, which occurs between 1000 and 3000 °C.^8^

Hydrometallurgical processing has already been discussed in the literature. However, a recycling process that provides complete treatment from cells to products while avoiding manual steps is still lacking. Previous studies on LFP battery recycling report manual dismantling of the batteries to separate the cathode active material by scraping,^9–11^ which is not feasible on an industrial scale. The main difference is that comminuted batteries form a complex mixed system containing internal materials such as metallic Al, Cu, plastics, graphite, and LiFePO_4_. Common hydrometallurgical approaches do not perform well with this type of mixed material. For example, the addition of H_2_O_2_ as a reducing agent during leaching results in selective Li solubilization, leaving behind a mixture of FePO_4_ and graphite that cannot be separated without calcination steps. This approach also causes metallic Cu losses during leaching and requires further purification steps, thereby increasing process costs and reducing overall attractiveness.^12^ In addition, deepened Fe removal should be obtained with solvent extraction, but wastewater contamination by toxic and persistent organic pollutants concerns the effluent treatment.^13^

Herein, we developed a new route for LFP battery recycling focusing on hydrometallurgy with lower energy demand than pyrometallurgical processes. The main recycling steps used 25 °C (leaching, aluminum (Al) precipitation, and ion exchange), and the maximum temperature was 90 °C in iron (Fe) precipitation and Li precipitation.

Real spent LFP batteries were comminuted and further separated by physical techniques to produce a cathode-rich material for leaching with H_2_SO_4_. Physical processing allowed battery solvent recovery for further usage by coupling exhaustion and coalescence filtration in the mill. Absence of H_2_O_2_ during leaching avoided the leaching of current collectors such as copper (Cu) and Al metal and reduced the reagent consumption. Alternatively, the consumption of H_2_O_2_ was redirected to promote the oxidative precipitation of Fe^2^^+^/Fe^3^^+^. No solvent extraction was used, which represents a low number of persistent toxic pollutants in the process effluent. Purification steps of the real leach solution with precipitation steps and an ion exchange process with low energy consumption (25 °C) were adopted for remaining Fe removal and further precipitation of Al and Li.

## 2 Results

Characterization of two cells, weighing 193.5 ± 1.2 g, involved dismantling to separate their components. Figure S5 depicts the LFP internal structure, showing the separate components after the dismantling process and the mass percentages. The cell was assembled around the cathode, separator, and anode along the central axis, with a separator positioned between the positive and negative electrodes to prevent short circuits.

LFP cathode scraped samples were analyzed by X-ray diffraction (XRD), as depicted in Figure S6, indicating the presence of LiFePO_4_ coated with C. Figure S7 depicts the XRD pattern for the anode, indicating the presence of graphite carbon at the active material.

Table S8 summarizes the chemical elements analyzed in the cathode and anode (Li, Fe, Al, Cu, and C), which were further calculated to the mass fraction in the cell. Cathode black powder is composed of LiFePO_4_ coated and conductive carbon bonded in an Al foil. From the cathode composition, the characterized LFP cells were composed of 6.6 ± 0.2% of Fe, 0.7 ± 0.2% of Li, and 9.0 ± 0.7% of Al. From the anode, the mean values were 0.1 ± 0.1% of Li, 14.9 ± 0.5% of Cu, and 11.6 ± 0.4% of C.

Cathode accounted for 36.5 ± 0.6% of the total mass of the cell, and the anode accounted for 28.8 ± 0.8%. Considering the chemical analysis, the active material from the cathode (LiFePO_4_.C) was 27.5 ± 0.7%, while from the anode, it was 13.8 ± 0.5%. Internal polymers were attached to the battery casing and served to protect the cathode and anode from direct contact with the aluminum case, thereby preventing short circuits. The separator was the polymer with the highest mass fraction in the cell (8.3 ± 0.1%), followed by the center axis (4.1 ± 0.2%) and the internal polymers (1.2 ± 0.2%). The remaining fraction corresponded to the aluminum case, which comprised 15.7 ± 1.0% of the total mass. The organic solved quantified by mass loss after drying resulted in 5.4 ± 0.6%.

Physical treatments were conducted to increase the metal content in the leached material. Discharged spent LFP cells were processed following the flowchart indicated in Figure S1. Active material, graphite, and metallic foils with less than 2 mm passed through the separation steps (61.4% of the initial mass), and metals were concentrated in comparison with the initial spent cells, Table S1. Metals concentration was due to 97% of the plastic materials removal in the retained material. No active material losses were reported in physical processes, but 14.7% of graphite was lost during milling. In addition, 19% Al foil and 30% Cu foil were retained. The organic solvent was released from the spent cells during milling, and 5.4% of the mass loss was due to solvent evaporation.

Leaching with H_2_SO_4_ was carried out without a reducing agent (H_2_O_2_, commonly reported in the literature). Figure 1A depicts results for the solid/liquid ratio effect (S/L ratio) and indicates that at a 1/5 S/L ratio, the LFP active material achieved a high leaching yield, with leaching percentages of 99.7% of Li and 99.6% of Fe. The low oxygen activity at 90 °C and H_2_O_2_ absence in leaching favored avoiding Cu leaching and the reduction of Al leaching. The leaching pattern with H_2_SO_4_ concentration variation is shown in Figure 1B. At 1.0 mol/L, Li, Fe, and Al reached leaching percentages of 98.8, 98.2, and 60.2%, respectively. In moderate acid concentrations (considered less than 1 mol/L), H^+^ consumption resulted in poor Fe and metallic Al leaching.

**Figure 1:**
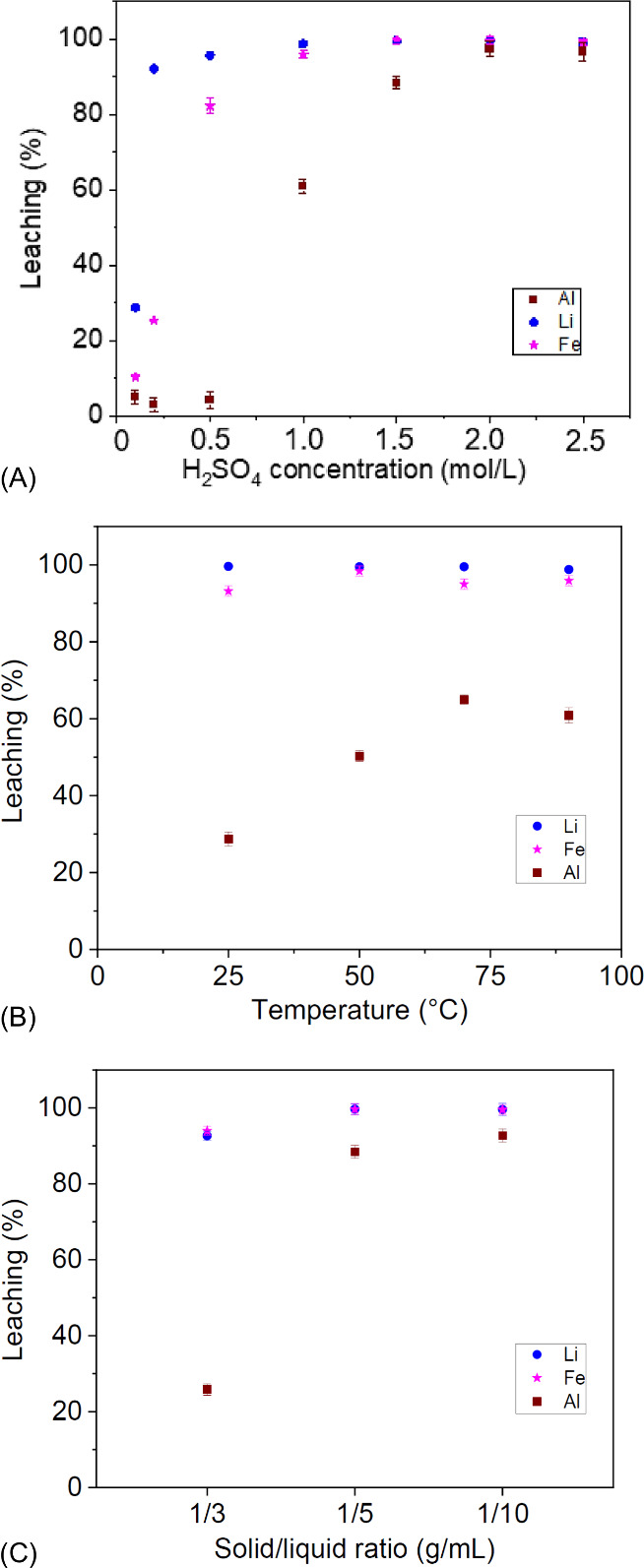
Leaching efficiencies of Fe, Li, and Al, varying the (A) S/L ratio with the conditions H_2_SO_4_ = 1.5 mol/L and *T* = 90 °C, (B) H_2_SO_4_ concentration with the conditions S/L ratio = 1/5 and *T* = 90 °C, and (C) temperature with the conditions S/L ratio = 1/5 and H_2_SO_4_ = 1 mol/L.

Figure 1C depicts the temperature variation with a H_2_SO_4_ concentration of 1 mol/L and an S/L ratio of 1/5. It was observed that the temperature has almost no effect on active material leaching. The leaching of Li remained at 99% in all temperatures evaluated, and the Fe leaching percentage remained around 95.6%. Temperature had a significant impact on metallic Al leaching, with an Al leaching of 28.7% at 25 °C, increasing to 60.5% at 90 °C. The final concentrations in leaching were 20 g/L Fe, 8 g/L Al, and 3 g/L of Li.

Fe removal was the first step in the proposed hydrometallurgical route, combining Fe precipitation and ion exchange with chelating resin. Tables S4 and S5 in the extended data depict parameters and results for Fe purification. H_2_O_2_ addition (0.8 V) and Na_2_CO_3_ pH correction (pH 3), within 120 min, achieved 93.5% Fe removal. Despite that, the Al and Li coprecipitations were 47.7 and 13.1%, respectively. Coprecipitation was reduced by increasing temperature and adding a solution of Na_2_CO_3_ (Fe.2 and Fe.3). Temperature was the main contributor, reducing coprecipitation to 3.0% of Li and 37.2% of Al losses with 96.0% of Fe precipitation. Na_2_CO_3_ solution improved to 92.9% Fe precipitation, and Li and Al losses were 0.5 and 35.5%, respectively.

Prolonged H_2_O_2_ reaction time and precipitation time (tests Fe.4 and Fe.5, respectively) aimed to reduce Li and Al losses, but coprecipitation of Al increased to 58.9% in test Fe.4 and 45.6% in test Fe.5. Further, 600 min for H_2_O_2_ time reaction raised the Li loss to 8.7%. The pH reduction to 2.0 (Fe.6) had no influence on Fe precipitation, but Li and Al coprecipitations were the lowest among the studied conditions (32.2 and 0.1%, respectively). Under studied conditions, the best parameters for Fe precipitation were pH 2.0, 80 °C, 120 min precipitation, and addition of Na_2_CO_3_ solution (test Fe.6). This condition combined selective Fe precipitation from Al (2.4 of Fe/Al mass ratio) and lower Li loss during precipitation (0.1%).

After reducing the Li and Al content during the precipitation step, the purity of the final product was determined by ICP-OES. The analysis indicated that the material contained 33% Fe, which was assumed to be present as FePO_4_·2H_2_O, since no crystalline phases were detected in the XRD of the obtained product. Based on molar mass calculations, the Fe content corresponds to approximately 62.9% FePO_4_·2H_2_O.

Residual Fe adsorption with aminophosphonic acid chelating resins (AP) was evaluated by parameters resin mass/solution volume (R/S), pH, and continuous/batch processes. First, tests in batch were carried out with R/S 0.04g/mL and pH 2.0. The Fe extraction at pH 2 was 40.8%, while at pH 3.0, it achieved 54.7%. At pH 3.0, Al and Li increased extractions to 24.7% and 6.8%. At 0.14g/mL of R/S, metals adsorption increased to 73.2% of Fe, 34.0% of Al, and 17.7% of Li.

Breakthrough curves for continuous ion exchange are indicated in Figure S2 for R/S 0.06 g/mL (equivalent to 10 mL bed volume) and Figure S3 for R/S 0.14g/mL (equal to 20 mL bed volume). In Figure S2, the concentration of outlet solution increased rapidly from 30 to 120 min, indicating that after one bed volume treatment (30 min), the resin achieved the breakthrough-point for Fe. Adsorption efficiency was close to zero after 120 min (*C*/*C*_0_ = 1), and the total adsorbed Fe mass was 35 mg, which represents only 30% of the initial Fe in the solution. The total resin capacity was 3.5 mg/mL of resin, and the mass-transfer zone was limited to 80 mL of solution or 8 bed volumes.

In Figure S3, the Fe mass-transfer zone was extended, and Fe was still under adsorption after treating 160 mL of solution (8 bed volumes). The total Fe extraction during the process was 100 mg, which represents 70% of Fe adsorption through the column. The total resin capacity for Fe adsorption was 5 mg of Fe/mL of resin. For the first 30 min, there was Li and Al adsorption *C*/*C*_0_ = 0.6, which stopped after two bed volumes or 60 min (*C*/*C*_0_ = 1). Despite the Li and Al adsorption during the first 30 min, the mass balance between feed and outlet solution indicated that neither metal has been extracted.

After Fe removal, the batch elution using a 3 M HCl solution resulted in 100% re-extraction of Fe and 100% re-extraction of Al. Li was not detected in the HCl eluate, remaining below the detection limit of 0.2 mg/L. The absence of Li in the eluate indicates that no Li extraction occurred during the Fe extraction step in the ion exchange columns.

The ion exchange process generated two process streams: (i) a purified solution, considered the main product, containing 4.8 g/L Al and 2.2 g/L Li, which was directed to the next purification step (Al precipitation); and (ii) an eluent stream, consisting of 3 M HCl containing 400 mg/L Fe and 40 mg/L Al.

Al precipitation after Fe removal produced Al­(OH)_3_. By adding the solid Na_2_CO_3_ until pH 5.0 and 80 °C (Al.1), the Al precipitation was 71.1%, and by adding 1 mol/L Na_2_CO_3_ solution (Al.2), the precipitation percentage was 70.7%. Li precipitation reached 24.8%, probably due to the PO_4_^–3^ ions remaining in solution.

Reducing the temperature to 25 °C (Al.3), 78.5% Al removal was achieved, and Li coprecipitation was reduced to 12.0%. At pH 6, Al­(OH)_3_ precipitation reached 95.8% (Al.4), and Li precipitation was 6.3%.

Li precipitation has been performed after Fe and Al removal in the aforementioned steps to obtain the Li_2_CO_3_ product. Due to high Li solubility at 25 °C, the precipitation must occur in elevated temperatures (80 °C), and the initial Li concentration in solution needs to be suitable for Li_2_CO_3_ supersaturation.^14^

The Li initial concentration was evaluated using synthetic solutions with concentrations of 1, 2, 3, and 4 g/L at pH 10 to simulate the real solution after Al removal. In the concentration of 1 and 2 g/L (Li.1 and Li.2), stoichiometric addition of Na_2_CO_3_ at pH 10 and 80 °C did not precipitate Li. Results showed that precipitation started at 3 g/L and achieved 91.3% of Li precipitation at a 4 g/L initial concentration. Then, for Li_2_CO_3_ saturation and precipitation in a real solution, the initial Li concentration must be 4 g/L.

Process mass balance in Figure 2 outlines a process for the recycling of spent LFP cells by hydrometallurgy. Outlet streams mass percentages for plastics, metallic foils (Al and Cu), and active material (Li and Fe) were indicated in each output. Discharging spent LFP cells provided energy for recycling. Physical treatments resulted in 97% of plastic retention, and part of metallic Al and Cu in retained material, 18.8 and 30% respectively. No active material losses were reported in the physical process, and 14.7% of graphite fines were lost in milling. Leaching residue was rich in C, Al, and Cu, allowing for the recovery of metallic foils without other chemical steps.

**Figure 2:**
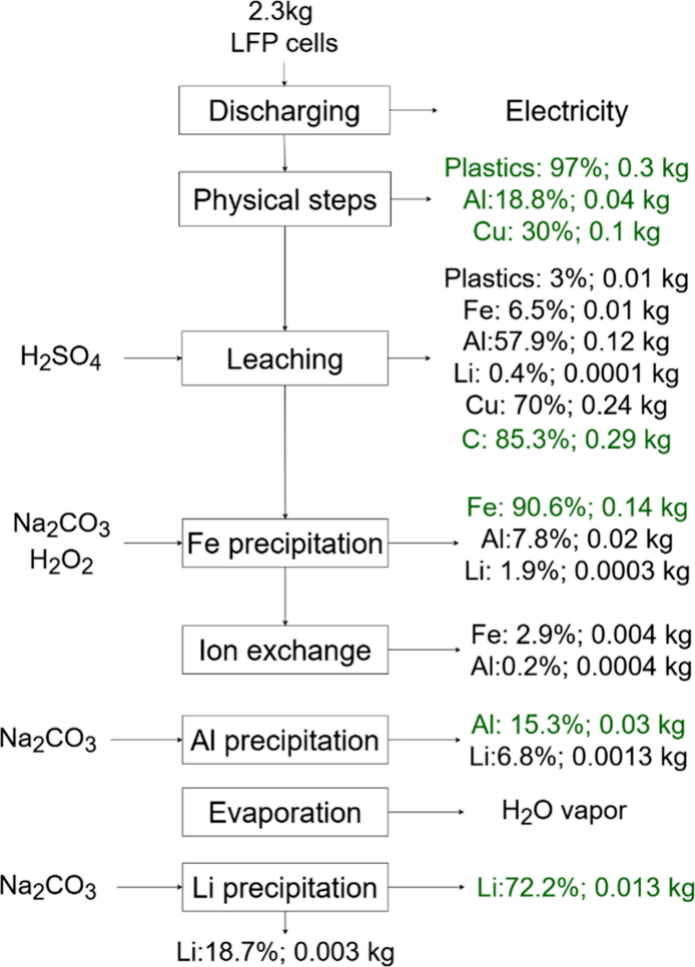
Mass balance for Li, Fe, C, Cu, and Al in the overall steps for hydrometallurgical recycling. The flowchart indicates the mass percentages of each material outlet streams. Percentages of Al, Cu, C, Li, and Fe are expressed relative to the total element mass. Each green label indicates the recovery values obtained during the LFP recycling process.

At pH 2.0, iron precipitation achieved 90.6% removal as iron phosphate. Despite that, it also favored AlPO_4_ precipitation, leading to 7.8% of Al loss and 1.9% of Li coprecipitation. The resulting solution passed through an ion exchange column with AP resin at pH 2.0, a 2 BV/h flow rate, and an R/S of 0.14g/mL. The first ion exchange column removed 1.6% of the initial Fe in the process, and a second column removed the residual 1.3% of Fe. The Al adsorption percentage was 0.8%. The Li adsorption was below the FAAS detection limit of 0.2 mg/L. This detection limit is sufficient to ensure that Li extraction remained below 0.01% in the recycling process; therefore, Li was disregarded in the mass balance.

After the Fe removal, the precipitation at pH 6.0 recovered 15.3% of Al by precipitating Al­(OH)_3_ with 6.8% of Li coprecipitation. After this step, the final Li concentration was 2 g/L, and to achieve Li supersaturation and precipitate Li_2_CO_3_, it was necessary to evaporate the solution. Then, precipitation at pH 10 and 80 °C recovered Li_2_CO_3_ with 72.2% efficiency. After precipitation as carbonate, about 18.7% of the overall Li remained in solution due to the Li concentration decline.

Table S2 in the Supporting Information provides a detailed separation of which streams were considered as recoveries and which ones represent losses to the mass balance. For example, for lithium (Li), the initial mass in the LFP cells was 0.018 kg (obtained from the metal fraction in LFP cells presented in Table S1), corresponding to 100% of the input. The retained solids in the physical separation and column raffinate steps did not contain any Li, under the detection limit of 0.2 mg/L of Li. The precipitated Li_2_CO_3_ product contained 0.013 kg of Li, representing 72.2% of the initial input. Li losses were attributed to the Li that was not leached from graphite (0.4%) and to undesired coprecipitation during the Fe and Al precipitation steps, accounting for 1.9 and 6.8%, respectively. Closing the mass balance, the residual Li remaining in solution after precipitation amounted to 0.003 kg (18.7% of the entry).

The XRD pattern of the produced Li_2_CO_3_ indicated that the obtained product should be an intermediate product, including the Na_2_SO_4_ phase as the main contaminant (Figure S8). Differing from the Li_2_CO_3_, the FePO_4_ product presented an amorphous XRD pattern, which was already discussed in the literature.^10^ The FePO_4_ and Li_2_CO_3_ products were also characterized throughout ICP-OES analysis with results in the Supporting Information (Table S10). Chemical analysis corroborates 1.8% of sodium contamination in Li_2_CO_3_. By cross-checking XRD data, chemical analysis, and molar calculations, the intermediate product was composed of 94% Li_2_CO_3_ and 6% of anhydrous Na_2_SO_4_. The Li:Na molar ratio was also calculated and found to be 32.4.

During the recycling process, the main reagents consumed were H_2_SO_4_, Na_2_CO_3_, H_2_O_2_, HCl, and ion exchange resins. The consumption was calculated and for each kilogram of LFP cells in the feed, 0.3 kg of H_2_SO_4_, 0.28 kg of H_2_O_2_, 0.047 kg of Na_2_CO_3_, 0.72 kg of ion exchange resins, and 0.57 kg of HCl were consumed.

## 3 Discussion

Previous authors^15^ reported that the LFP battery has anode mass fractions of 10% Cu foil and 13% anode active material, which seems to be close to the percentages presented in Tables S7 and S8. The active material in the cathode represents 27.5 ± 0.7%, and Al foil 9.0 ± 0.7%, while other authors indicated 6% Al foil and 25% active material in the LFP battery.^15^ Despite similar values between the characterized battery and the literature, cells should have several configurations and fractions once different companies are involved in battery manufacturing.

Graphite is the most common anode component because it has high electrical conductivity, low cost, a mature production process, and abundant resources.^16^ The presence of C in the cathode indicates the conductive carbon coating for performance improvement.^17^

Discharging with electrical resistance demonstrated the possibility of energy reuse and provided hydrothermal heating for Fe and Li precipitations. Milling released 5.4% of cell mass loss due to organic solvent volatilization. In industrial facilities, the volatilized solvent must be recovered by coupling exhaustion and a coalescence filter. The combustion of LIB electrolytes releases toxic gases mixtures (H_2_, CO, CO_2_, CH_4_, C_2_H_4_, C_2_H_6_, C_3_H_8_, HF, POF_3_, PF_5_, ethyl fluoride, and propylene).^18^

During milling, the organic solvent evaporated and was not handled. Managing the electrolyte involves industrial approaches such as installation of exhaust systems in the mill to capture volatile compounds, installation of coalescence filters, or condensation systems at the end of the exhaust line for recovery, or, ultimately, combustion of the electrolyte. The choice of an appropriate industrial approach for electrolyte management requires an economic feasibility analysis in pilot-scale units, which is beyond the scope of this study.

Retained materials from physical processing, such as plastics and metallic Al from external protection of the cells, were sent to appropriate recyclers. End-of-life plastics represented 9% of greenhouse gas emissions from the plastic life cycle in 2015, and incineration was the largest emission share.^7^ Li-batteries recycling that includes plastics recovery and recycling contributes to the reduction of CO_2_ emissions from incineration and polymer fabrication.

Graphite losses are common during milling processes such as spheroidization, and fines formation yields only 30–50% of graphite recovery.^19^ Despite that, 85% of graphite was recovered in leaching residue, contributing to an expressive CO_2_ footprint reduction. By considering each 100mton of LFP batteries processed, 12.8 tons of graphite must be recovered, and consequently, 46.9 tons of CO_2_ emissions must be saved. In addition, graphite produced after leaching must be a material source for high-value products such as graphite oxide and graphene.^20^

The most common reductor for LFP battery leaching is H_2_O_2_,^21^ which would act as an oxidizing or reducing agent, depending on the pH and Eh conditions. In general, H_2_O_2_ acts by oxidizing Fe^2+^ to Fe^3+9^, leading to selective Li leaching (eq 1). Despite that, in industrial facilities, LIBs must be physically processed to obtain a mixture of cathode active materials, graphite, and Al and Cu foils. In this case, Li selective leaching increases reagent consumption by reacting with Al and Cu foils.

$$2{{\mathrm{LiFePO}}_{4}}_{\left(s\right)}+H_{2}{{\mathrm{SO}}_{4}}_{\left(\mathrm{aq}\right)}+H_{2}{O_{2}}_{\left(\mathrm{aq}\right)}\to 2{{\mathrm{FePO}}_{4}}_{\left(s\right)}+{\mathrm{Li}}_{2}{{\mathrm{SO}}_{4}}_{\left(\mathrm{aq}\right)}+2H_{2}O_{\left(\mathrm{aq}\right)}$$ 1

Insoluble Cu species are predominant in acid media, redox potential lower than 0.5 V, and temperature 90 °C (Figure S4 in extended data), but H_2_O_2_ use must favor the metallic Cu leaching in H_2_SO_4_.^3^ Copper leaching hinders the purification process due to precipitation at pH 5–6, leading to a mixture of Al­(OH)_3_ and Cu­(OH)_2_. Further, Cu^2+^ ions extraction at the ion exchange columns may reduce the efficiency of Fe^3+^ extraction.

Equation 2 indicates the chemical reaction of active material leaching by H_2_SO_4_, while eq 3 indicates the chemical reaction of Al foil.

$$2{{\mathrm{LiFePO}}_{4}}_{\left(s\right)}+3H_{2}{{\mathrm{SO}}_{4}}_{\left(\mathrm{aq}\right)}\to 2{{\mathrm{FeSO}}_{4}}_{\left(\mathrm{aq}\right)}+{\mathrm{Li}}_{2}{{\mathrm{SO}}_{4}}_{\left(\mathrm{aq}\right)}+2H_{3}{{\mathrm{PO}}_{4}}_{\left(\mathrm{aq}\right)}$$ 2

$$2{\mathrm{Al}}_{\left(s\right)}+3H_{2}{{\mathrm{SO}}_{4}}_{\left(\mathrm{aq}\right)}\to {\mathrm{Al}}_{2}({\mathrm{SO}}_{4}{{)}_{3}}_{\left(\mathrm{aq}\right)}+3{H_{2}}_{\left(g\right)}$$ 3

Active material was leached with an S/L ratio greater than 1/5, achieving Li and Fe leaching percentages of 99.7 and 99.6%, respectively. The S/L ratio and H_2_SO_4_ concentration impact on acid availability and contribute to ion diffusion, favoring solid dissolution into the aqueous phase.^11^ The literature reported that an S/L ratio of 1/3 to 1/10 increases LiFePO_4_ leaching in H_2_SO_4_.^10,11,22^

Despite the fact that the same acid is necessary to leach metallic Al or LiFePO_4_ (eqs 2 and 3), results indicated that LiFePO_4_ leached better in poor conditions. At a 1/3 S/L ratio, the LiFePO_4_ cathode leached more than 90%, while metallic Al leached 26%. Under these conditions, 0.12 mol of H_2_SO_4_ was necessary for LiFePO_4_ and Al acid leaching, but only 0.08 mol of H_2_SO_4_ was fed into the reactor. In the lack of H^+^, the active material powder reacted faster than metallic Al due to the higher surface contact and reaction kinetics.^11^

H_2_SO_4_ concentrations higher than 1 mol/L resulted in active material leaching (98.8% of Li and 98.2% of Fe) and metallic Al leaching 60.5%. The lack of acid in lower concentrations favored faster reaction kinetics of the active material. Temperature improved the poor reaction kinetics of metallic Al, leading to a strong dependence and reducing it to 28.7% at 25 °C.^23^ corroborated that metallic Al dissolution is favored by temperature, with 90% Li–Fe–P leaching and 22.5% Al collector at 20 °C. Steadily LiFePO_4_ and lower metallic Al leaching efficiencies at 25 °C were the main assumptions to select leaching parameters 1 mol/L H_2_SO_4_, S/L ratio 1/5, and 25 °C.

The reagent used for neutralization during purification was Na_2_CO_3_. Others, such as CaO and Ca­(OH)_2_, form insoluble products such as CaCO_3_ and Ca_2_SO_4_,^24^ while large volumes of NaOH bled into the suction side of pumps or hinder the product filtration.^24^

Precipitation in aqueous media is controlled by *G*_f_°. The pH, redox potential, ionic activities, and temperature influence the formation of insoluble species, and the Pourbaix diagram represents the species formed between lines in the diagram (Figure 3).

**Figure 3:**
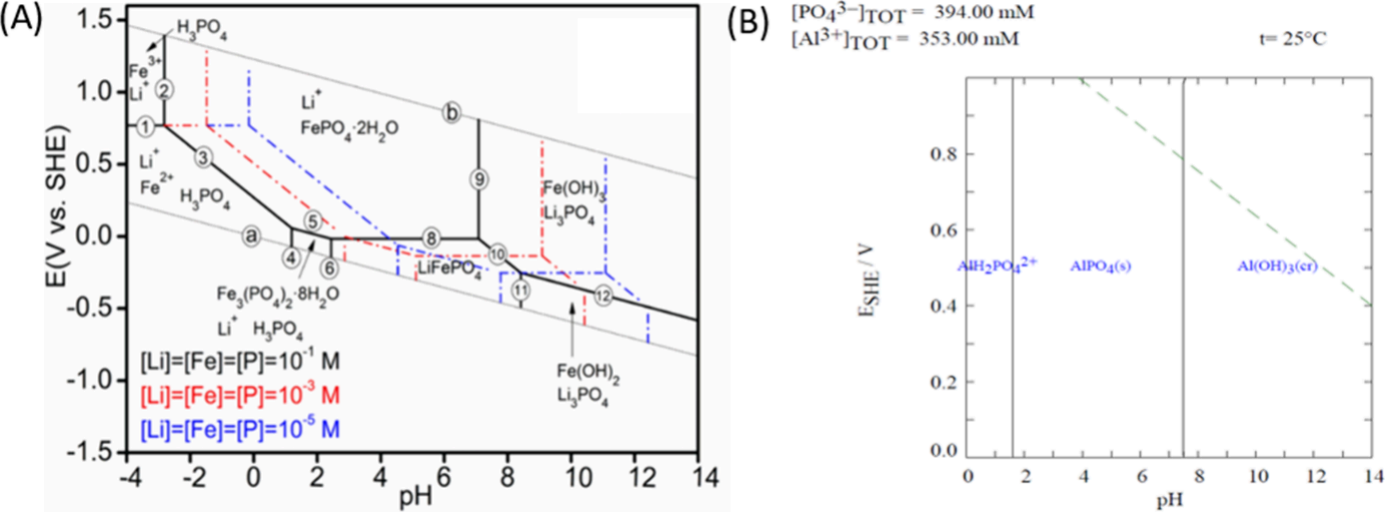
Pourbaix diagrams for the (A) Li–Fe–P–H_2_O 25 °C^25^ and (B) Al–P–H_2_O systems at 25 °C, illustrating the predominant species (Simulated with Hydra-Medusa).

Figure 3A shows that Fe^3+^ is preferentially precipitated at pH 2 and redox potentials above 0.5 V in the form of FePO_4_·2H_2_O. Fe^2+^ to Fe^3+^ oxidation was carried out by adding 7% (v/v) H_2_O_2_ (eq 4), raising the leaching liquor redox potential to 0.8 V.

$$2{\mathrm{Fe}}^{2+}+H_{2}O_{2}+2H^{+}\to 2{\mathrm{Fe}}^{3+}+2H_{2}O$$ 4

During Fe precipitation, impurities such as Al^3+^ and Na^+^ (the last added during neutralization) may contaminate the precipitate.^23^ In particular, Al^3+^ in phosphate leaching solutions may precipitate in different amorphous forms of AlPO_4_ at pH 3 ^26^ or precipitate as Al­(OH)_3_ from pH 3.5, making it difficult to separate Fe and Al products.^27^ In addition, the Fe–Al precipitate amorphous structure resulted in Li^+^ ions being trapped by coprecipitation.^28^ Coprecipitation was avoided by enhancing the Li^+^ ions' kinetic energy with temperature,^27^ which resulted in 0.1% of Li coprecipitation at 80 °C.

Chelating resins with functional group aminophosphonic (PuroliteS950) were tested due to their selectivity order for Fe as Fe^3+^ > Cu > Al^3+^ > Ni > Co^2+^,^29,30^ and efficient metal adsorption in acid media.^31,32^ Resins contain three types of active centers: phosphonic acid (−POH), hydroxyl acid (−OH), and amine (−NH−) functional groups (eq 5).

$${\mathrm{HSO}}_{4}^{-}+R-\mathrm{NH}-\mathrm{PO}(\mathrm{OH}{)}_{2,r}⇄R-({\mathrm{NH}}_{2}^{+}{\mathrm{HSO}}_{4}^{-})-\mathrm{PO}(\mathrm{OH}{)}_{2,r}$$ 5

The sorption of transition elements proceeds via coordination bonds with phosphonic sites, or by ionic interactions with the hydroxyl and amine groups.^33^ Chelation is chemically favorable to occur with Fe^3+^, while weaker ionic interactions may occur with lower-dimensional atoms such as Li. The higher Li and Al concentrations at this step favored the formation of the weaker ionic interactions with the resin. In batch tests were observed poor selectivity extraction of Fe. At pH 2.0, H^+^ competition with metallic ions for the resin active sites increased selectivity. The Fe/Al ratio extracted was 0.31 at pH 2 and 0.27 at pH 3, indicating that pH reduction favored the Fe extraction. The lower Li and Al chelation affinity with aminophosphonic sites was feasible to avoid in column tests, once the continuous process usually results in better selectivity.^30^

The breakthrough curve depicted in Figure S2 indicates that the concentration of the outlet solution increased rapidly from 30 to 120 min. Resin saturation started after 30 min (one bed volume), and the fixed bed started to be ineffective for extraction after 120 min (*C*/*C*_0_ = 1), leading to a narrow mass-transfer zone. The total adsorbed Fe represented only 30% of the initial Fe in solution.

By increasing R/S to 0.14 g/mL (bed volume = 20 mL, Figure S3 ), the Fe mass-transfer zone was extended once more available active resin sites were increased. The difference between the Fe break-point time and ineffective-bed time was higher, and Fe was still under adsorption at 180 min. The total Fe extraction represented 70% through the first column, and the solution was passed through a second column to achieve 100% Fe extraction. Li and Al extraction achieved *C*/*C*_0_ = 1 at one bed volume, and the total Al extracted was 0.9% with no Li loss. The continuous ion exchange was necessary to improve selectivity. Results obtained match with literature reports of better selectivity in columns to remove Fe^3+^ from acid liquor.^34^

The use of aminophosphonic resins for Fe extraction may face challenges in industrial applications. Two critical aspects are the possibility of resin regeneration and the consumption of reagents during this process, as well as the final disposal of nonfunctionalized (spent) resins. The literature reports that aminophosphonic resins can operate under acidic conditions;^35^ however, the long-term stability and performance of these resins must be further investigated in pilot-scale units.

Al precipitation of 78.7% at pH 5 was related to the injection of oxidizing agent (H_2_O_2_) in Fe precipitation and the selected pH. Reference 36 has precipitated Al from a mine drainage solution with H_2_O_2_ addition and pH 5.5, resulting in a lower Al average precipitation of 76.3%. Li coprecipitation was not avoided at higher temperatures, which were higher at 80 °C (24.8 and 24.2%). Further, the lower influence of reagent type in solid or solution suggests that the Li was not coprecipitated with Al­(OH)_3_, and the Li precipitation is due to the remaining PO_4_^3–^ ions in solution. Reference 37 describes the same pattern in a system of Li_2_SO_4_ and Na_3_PO_4_; as the temperature was increased to 70 °C, 90% of Li_3_PO_4_ precipitation was obtained.

After Al precipitation, insoluble Li species like Li_2_CO_3_ and Li_3_PO_4_ would precipitate by the addition of reagents Na_2_CO_3_ or Na_3_PO_4_ above pH 8.^9,10,12^ The synthetic solution study indicated that Li precipitated as Li_2_CO_3_ at an initial Li concentration of 4g/L, a condition that could not be achieved in the real leaching solution without evaporating 50% of its volume. At pH 10, 72.2% of Li precipitated, while 18.8% remained in solution. The literature corroborates that Li_2_CO_3_ was not completely precipitated even with a 35 g/L initial concentration from LiCl solution, after 8 h reaction and 60 °C,^38^ indicating that Li would remain in the solution and must be recirculated to increase Li recovery.

Lithium concentration for precipitation was demonstrated in this work; however, the evaporation of acidic solutions at 100 °C for 2 h presents significant challenges for industrial applications. Evaporation is energy-intensive and reduces the overall feasibility of the process on an industrial scale. As an alternative, membrane-based processes may be used to concentrate lithium-bearing solutions from the proposed process. Electrodialysis, ultrafiltration, and nanofiltration offer more energy-efficient options; in particular, electrodialysis has gained increasing interest for the separation of lithium-ion battery recycling processes. Its application to the concentration of lithium-containing solutions has also been reported in the literature.^39,15^

In this regard, the process discussed contributes to improving lithium recovery through the recycling of LFP batteries. The process focuses on maximizing the recovery of both lithium and iron, while also maintaining the recovery of aluminum and copper metallic foils, as well as graphite. The Li_2_CO_3_ produced should be considered an intermediate product, a common result for the first precipitation step. The Li produced should be further explored in solubilization and second precipitation steps to improve purity and application in the battery market.

The remaining solution from the lithium precipitation step, which contained 18.7% of the total lithium from the LFP cells input, could serve as an additional source for lithium recovery. In industrial facilities, intermediate products and effluents containing concentrated solutions are typically recirculated to improve the overall recovery. In this scenario, the total lithium recovery, initially calculated at 72.2%, could increase to up to 90.9% when using this solution. Nevertheless, the recirculation of concentrated salt solutions still requires further validation, as other purification steps may be needed to reduce the Na content and concentrate Li.

The final Li_2_CO_3_ product was reported to be a 32.4 molar ratio between Na and Li. This was equivalent to the purity of compounds 94% Li_2_CO_3_ and 6% anhydrous Na_2_SO_4_. This specification does not meet the technical grade, reported in literature to be 99%, or the battery-grade reported as 99.5% of Li_2_CO_3_.^40^ Despite that, recycling processes must be a combination of purification steps, and intermediate products are commonly produced before achieving battery-grade materials. The refining of Li_2_CO_3_ should be obtained by solubilization of intermediate products and precipitation with CO_2_.^41^

In pyrometallurgical processes for lithium-ion battery recycling, smelting operations typically employ temperatures above 1200 °C.^4^ Maintaining such temperatures requires a large energy input, and smelters are associated with significant greenhouse gas emissions. In addition, the PVDF binder decomposes at approximately 475 °C, releasing HF and short-chain hydrocarbons.^42^ At temperatures above 1300 °C, lithium is largely lost to the slag phase.^43^ Although the energy consumption of laboratory-scale equipment cannot be directly extrapolated to industrial units, eliminating high-temperature furnaces implies a substantially lower thermal-energy demand and a reduced CO_2_ footprint compared with pyrometallurgical recycling.

Safety considerations are essential when discussing the hydrometallurgical process developed in this study. Cell characterization requires mechanical dismantling, including the cutting and discharging of the cells. The organic solvents released during cell opening and milling must be handled appropriately in industrial facilities following established safety procedures. Finally, the acids and H_2_O_2_ used in the process must be handled safely and neutralized, as they constitute the main chemical hazards.

## 4 Methods

The received cylindrical spent LFP cells were discharged using an external resistive load. The cells were connected in series using copper wires, and the circuit was then connected to a resistive load. The batteries were discharged for 24h and subsequently tested to verify any residual charge.

Spent cells were manually dismantled by starting with a horizontal cut with a saw at the top of the cell. The first cut in the top of the cell allowed pulling the metal case with pliers, bearing inner components, and separating the Al case. The cathode, anode, and separator were involved in a plastic axis, forming a cylinder roll. The cylinder was unrolled manually for characterization, separating the electrodes and a polymer-based separator. After dismantling, each cell component was dried in a fume hood for 24h at room temperature. The mass fraction of each component was determined by dried mass weight in an analytical balance, and the dismantling process was done in duplicate.

The active powder has been analyzed in an Eltra elemental analyzer to determine carbon content in both the anode and cathode. About 50–100 mg of the sample were mixed with tungsten and iron accelerator in a predetermined proportion indicated by equipment method. The selected method was the same for carbon content determination in cement, and analyses were performed in triplicate.

Samples of cathode and anode (1 g) were dissolved and separated in aqua regia, a 1/3 proportion mixture between nitric acid (HNO_3_ = 65%) and hydrochloric acid (HCl = 36.5%), respectively. The solid material was solubilized using a solid/liquid ratio (S/L) of 1/10, 60 °C heating, and 24 h reaction in a fume hood. After 24 h, the solution was filtered, and the carbon remained solid. The solution was diluted in HNO_3_ 3%, and the concentrations of Li, Fe, Al, and Cu were determined using a flame atomic absorption spectrophotometer (FAAS). The digestion process was performed in duplicate, while the FAAS analysis was conducted in triplicate. The calculated error for each metal considered the combined errors from sample duplication and the chemical analysis.

Comminution processes were carried out by two grinding (cutter mill) and two sieving steps until the desirable particle granulometry (lower than 2 mm). A cutter mill was equipped with removable grids (apertures 3 mm and 9 mm). Sieving steps were carried out with steel sieves (apertures 2 and 5.6 mm), a cover, and a background. Reference 44 reported the same grinding and sieving steps to achieve cathode liberation, metal concentration in passerby fraction, and increased leaching extraction. Before the grinding process, apertures in the knife mill were sealed, except for the feed and the exit apertures, avoiding active material losses.

The process started with 2.3 kg of LFP cells, which corresponds to 12 cell unities. Figure S1 depicts physical treatment steps performed on LFP batteries. The terminology selected was “P” for passing materials, “G” for retained grid fractions, and “S” for retained sieve materials. Equipped with a 9 mm grid, the material was grounded for 20 min and separated into two parts: G9 (retained in the 9 mm grid) and P1 (passing 1). P1 was sieved in a 5.6 mm stainless steel sieve, producing S6 (retained in 5.6 mm) and P2 (passing 2). After this process, P2 was ground in the cutter mill for 30 min with a 3 mm grid separating into two parts: G3 (retained in the 3 mm grid) and P3 (passing 3). P3 were sieved in a 2 mm sieve, leading to S2 (retained in the 2 mm sieve) and P4 (passing with a particle size lower than 2 mm).

After the process, the material obtained in P4 was homogenized and separated into 0.5 kg packages. Samples of comminuted material (10 g) were dissolved in aqua regia using a solid/liquid ratio (S/L) of 1/10, 60 °C heating, and 24 h reaction in a fume hood; after reaction, graphite was collected by filtration. The solution was diluted in HNO_3_ 3%, and concentration of Li, Fe, Al, and Cu was determined in flame atomic absorption (FAAS).

The designed hydrometallurgical process includes as main steps: batteries discharging; spent LFP batteries leaching; Fe precipitation; residual Fe extraction with ion exchange columns; Al precipitation; and Li precipitation.

Leaching experiments were carried out using ground LFP batteries with the composition of the solid material presented in Table S1 (extended data). The leaching procedure consists of a three-bottleneck flask with the desirable acid volume connected to a glass condenser plugged into a water bath under magnetic stirring and heating by a hot plate. The temperature was stabilized and measured before the reaction time with a glass thermometer plugged into a rubber stopper. After the reaction time, the solid/liquid mixture was filtered in a vacuum system to obtain a liquor for chemical analysis.

The solid residue after filtration was washed with ultrapure water immediately after filtration to avoid Cu solubilization. The residue was then dried at 60 °C for 24 h. Table S2 in the extended data summarizes the leaching experiments with H_2_SO_4_ varying solid/liquid ratio, H_2_SO_4_ concentration, and temperature. The leach solution and the leach residue solubilized in aqua regia were analyzed in flame atomic absorption (FAAS). The mass balance was calculated considering the amount of the metal in the leach solution and leach residue. Equation 6 was used to calculate the leaching percentages of each metal. Extraction percentage or leaching efficiency is the ratio of the metal mass in leach liquor to the sum of leach liquor and leach residue.

$$\mathrm{Extraction}\%=\frac{[Me_{l}]\times V_{l}}{[Me_{l}]\times V_{l}+[Me_{r}]\times V_{r}}\times 100$$ 6

Table S4 in the extended data shows the parameters studied for Fe precipitation, which were pH, temperature, solid/solution reagent type, precipitation time, and H_2_O_2_ time. For each test, 30 mL of the real H_2_SO_4_ leaching solution was mixed with 2.1 mL of H_2_O_2_ analytical grade (7%v/v). This procedure was carried out to raise the Eh to 0.8 V and Fe^2+^ oxidation (eq 4).

The first condition (Fe.1) was set to evaluate a wide proportion of Fe precipitation (above pH 2,0, 0.8 V, and 120 min of precipitation time – the center of FePO_4_.2H_2_O region in Figure 3). After the reaction time, the solution was filtered with a vacuum system, and metals in the solution were determined in FAAS. The solid retained in the filter was washed with ultrapure water at pH 3.0. Then, the solid was digested in aqua regia for 24h using an S/L ratio of 1/10 at 25 °C for mass balance.

Mass balance for all precipitation testes was calculated using eq 7. The precipitation percentage was the ratio between the mass of the target metal in the precipitate (*Me*_*p*_) to the sum of metal in the solution (*Me*_*l*_) and in the precipitate (*Me*_*p*_).

$$Me\;\mathrm{precipitation}\%=\frac{Me_{p}}{Me_{l}+Me_{p}}\times 100$$ 7

After precipitation, the solution was treated with an ion exchange resin for the remaining Fe removal. The parameters studied were pH, in batch, or continuous process (Table S5 in extended data). The resin was washed with 3.0 mol/L HCl solution and ultrapure water, in alternating washes, in Erlenmeyer flasks in an orbital shaker for 30 min and 200 rpm. The procedure was repeated two times to remove all impurities and to charge functional groups of the resin with exchangeable H^+^ ions. Then, the filtered resin was dried at 50 °C for 24 h.^45^

Tests carried out in batches varied two parameters: resin mass and pH. Each test was carried out with 30 mL of leaching solution from the Fe precipitation step. The dried resin was weighed in an analytical balance, and the pH was adjusted with solid Na_2_CO_3_. Experiments were carried out at 200 rpm and 25 °C for 2 h.

The experimental setup of the column is depicted in Figure 4. The continuous process was carried out with the best results in the batch to increase the Fe selectivity. The first bed volume was set to achieve the same ratio of the batch test (IX.Fe.3), which was 0.14 g/mL (resin mass to solution volume).

**Figure 4:**
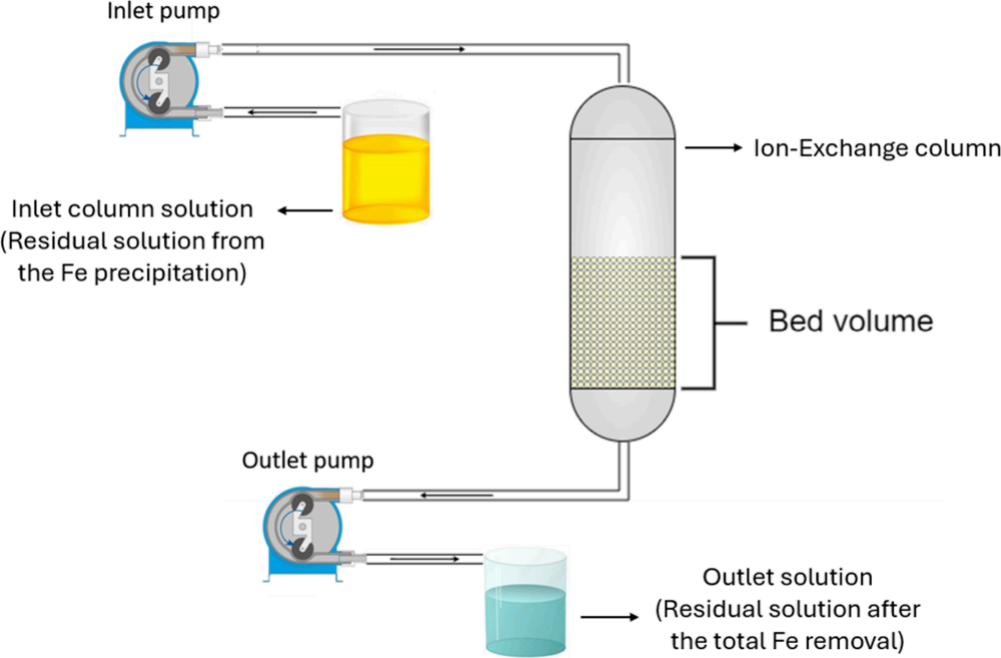
Schematic of the ion exchange system. “Inlet” indicates the battery leachate fed to the fixed-bed column; “outlet (raffinate)” denotes Fe-depleted solution; flow direction and bed volume are indicated.

Dried resins were hydrated with deionized water in an Erlenmeyer flask on an orbital shaker for 30 min and packed in a fixed bed volume (BV) of 10 (IX.Fe.5) and 20 mL (IX.Fe.6). The inlet and outlet pumps were set to maintain a flow rate of 40 mL/h throughout the column. This setup configuration maintained the flow rate at 40 mL/h for both tests, which represents 4 BV/h for a 10 mL bed volume and 2 BV/h for a 20 mL BV.

After the extraction of Fe in the columns, the resins were unpacked from the fixed-bed and eluted in batch using a 3 M HCl solution in Erlenmeyer flasks in an orbital shaker for 60 min and 200 rpm. The R/S ratio was maintained at 0.14 g of charged resin per milliliter of eluent. The filtered solution was analyzed by FAAS, and the resin regeneration was confirmed by a mass balance of the metals in the eluate and the solution after extraction.

The purified solution was forwarded to Al precipitation, and Table S5 in the Supporting Information shows the tests carried out for Al precipitation. Solid or 1.0 mol/L solution of Na_2_CO_3_ was added at room temperature or 80 °C under magnetic stirring, at pH 5.0 and 6.0. After the precipitation, the solution was filtered with a vacuum system for FAAS analysis. The solid was washed with ultrapure water at pH 5.0 and dried at 60 °C for 24 h.

Li precipitation experiments were first carried out with a synthetic solution prepared in ultrapure water, dissolving solid Li­(OH). To achieve the same pH and SO_4_^–2^ concentration, the pH was adjusted with concentrated H_2_SO_4_. Table S6 in the extended data shows the tests in a synthetic solution for Li precipitation with Na_2_CO_3_ to obtain Li_2_CO_3_. Concentration is one of the most important factors influencing the Li precipitation process, so solutions with Li concentrations of 1, 2, 3, and 4 g/L were prepared.^14^ Also, tests were carried out at 80 °C because of the lower solubility product of Li_2_CO_3_ at 80 °C (K_sp_ = 1.36 × 10^–4^) than at 25 °C (K_sp_ = 1.17 × 10^–3^). Li precipitation from a real leaching solution was done after an evaporation step to increase the Li initial concentration. Evaporation was performed on a hot plate at 100 °C and 2 h. Then, the precipitating agent was added at a pH of 10 at 80 °C and 120 min.

The process mass balance was calculated based on the elemental composition of the LFP cells. From the characterization data, the mass of each element in the feed stream was determined. After each purification step, metal losses and recoveries in the various streams were quantified according to their elemental compositions and compared with the feed. The results were presented in a flowchart showing the elemental mass percentages in the LFP cells. In addition, the supplementary data include the results for retained solids, precipitated products, column raffinate, Li-bearing solution, and the overall process losses.

## 5 Conclusions

The sustainable and circular life cycle of lithium-ion batteries depends on technological development to reduce the life cycle, CO_2_ footprint, and circularity of critical raw materials. By using physical pretreatments, H_2_SO_4_ leaching, and purification methods such as precipitation and ion exchange, our study presented a recycling process with lower energy demand (mainly at room temperature), recovering 97% of plastics and 85% of graphite materials responsible for high CO_2_ emissions in their entire life cycle. Organic solvents were evaporated during milling (5.4%) but were not degraded, opening the possibility to recover using industrial approaches such as exhaustion and condensation recovery. H_2_SO_4_ leaching without H_2_O_2_ resulted in no Cu leaching, especially due to leaching in a reducing environment (0.5 V). During the recycling, it was not necessary to address Cu contamination with other steps. The leaching provided 99% Li and 96% Fe leaching, depleting metals, and recovering graphite. The difficult Fe removal step in aqueous solutions was solved by combining precipitation and a low-energy-demand ion exchange process, removing Fe while avoiding solvent extraction steps and water pollution. The total Li_2_CO_3_ precipitation was 72.2%, and stream recirculation in industrial processes must increase Li recovery efficiency to 90.9%. Production of Li_2_CO_3_ from spent LFP batteries contributes to the reduction of the forecasted high volumes of hazardous waste generation and recycling supply of Li for electric vehicles by 2030.

## Supplementary Material



## Notes

Metal mass percentages in LFP cells and comminuted material; mass balance and metal composition in each stream of the LFP recycling process; summary of leaching parameters; Fe precipitation parameters and Fe/Li/Al precipitation results; ion exchange parameters and batch extraction results for Fe, Li, and Al; Al precipitation parameters and Li/Al precipitation results; Li precipitation results at varying initial Li concentrations; physical processing streams and mass balance; breakthrough curves for Li, Fe, and Al adsorption in Purolite S950 (continuous process; bed volume 10 mL); breakthrough curves for Li, Fe, and Al adsorption in Purolite S950 (continuous process; bed volume 20 mL); Pourbaix diagram for the Cu–SO_4_–H_2_O system; dismantled LFP cell and mass percentages of each internal component; X-ray diffractogram of the cathode active material; X-ray diffractogram of the anode active material; elemental mass percentages in cells based on metal concentrations in cathode and anode; mass percentages of components in dismantled LFP cells; X-ray diffractogram of Li_2_CO_3_ precipitated at pH 10; and elemental mass percentages in FePO_4_ and Li_2_CO_3_ products (PDF)
