# Supplementary material for: Hydrometallurgical Strategy To Reduce Waste through the Recycling of Lithium Iron Phosphate Batteries
Source: ACS Omega. 2025 Dec 23;11(1):1019–29. doi: 10.1021/acsomega.5c07786 (PMC12809529; doi:10.1021/acsomega.5c07786)
Supplement: Supplementary file 1 [file ao5c07786_si_001.pdf]

Hydrometallurgical strategy to reduce waste through the recycling of lithium iron phosphate batteries

David da Silva Vasconcelos*[1]; Denise Crocce Romano Espinosa[1]; Jorge Alberto Soares Tenório[1]; Amilton Barbosa Botelho Junior[2]; Luciana Assis Gobo[3].

[1] Chemical Engineering Department, Polytechnique School, University of São Paulo, Reitoria Street, 374, Butantã, São Paulo, Brazil
[2] Department of Chemical Engineering, Norwegian University of Science and Technology, Trondheim, Norway
[3]TUPY S/A, Rua Albano Schmidt, 3400, Joinville, 89227-901, Santa Catarina, Brazil

*Corresponding author: david.vsc@usp.br

Abstract

Batteries with $LiFePO_4$ as active material stand out by the absence of critical materials such as nickel and cobalt, thermal stability, and security. In the next years, high volumes of LFP batteries will achieve end of life, and overall materials recover contribute to meet the Li demand and reduce CO_2 footprint. 97% of plastics and 85.3% graphite recovery avoided materials burning into furnaces and reduced CO_2 footprint from recycling. Leaching cathode active material using H_2SO_4 without H_2O_2 resulted in active material leaching with reduced metallic foils solubilization and less reagent consumption. Redirect H_2O_2 consumption to Fe removal by precipitation and combining with ion exchange columns at 25°C successfully deepened Fe purification from solution. Precipitation of Al recovered 15.3% as $Al(OH)_3$ coproduct. After evaporation in a real solution, 72.2% of Li was precipitated as Li_2CO_3, contributing to increasing the recycling share in Li supply.

Keywords: Electric vehicles, critical raw materials, $LiFePO_4$, CO_2 footprint, leaching, ion exchange, energy.

Supporting information for publication

Table S1: Comparation between the metals composition in mass percentage of strategic materials in LFP cells and comminuted material (P4 stream).

Element	Spent LFP cells (%)	Comminuted material (%)
Li	0.8	1.4
Fe	6.6	11.0
Al	9.0	11.9
Cu	14.9	17.0
C	14.9	20.7
Plastics	13.6	-

Table S2: Supporting table of mass balance, considering the metal composition in each stream of the LFP recycling process.

Stream	Li (kg)	Fe (kg)	Al (kg)	Cu (kg)	C (kg)
LFP cells (Entry)	0.018	0.152	0.207	0.345	0.343
Retained solids from physical separation	-	-	0.039	0.104	-
Precipitated products (Graphite, Li_2CO_3, $FePO_4$ or $Al(OH)_3$)	0.013	0.138	0.032	-	0.292
Column raffinate	-	0.004	0.0004	-	-
Bearing solution	0.003	-	-	-	-
Losses (Milling and precipitation losses, and residual in graphite)	0.002	0.010	0.136	0.242	0.050

Table S3: Leaching experiments carried out with H_2SO_4. Studied parameters: H_2SO_4 concentration, S/L ratio and temperature.

Test	H_2SO_4 concentration(mol/L)	Solid/liquid ratio(g/mL)	Temperature (°C)
S01.15.90	0.1	1/3	25
S02.15.90	0.2	1/5	50
S05.15.90	0.5	1/10	75
S10.15.90	1.0	-	90
S15.15.90	1.5	-	-
S20.15.90	2.0	-	-
S25.15.90	2.5	-	-

Table S4: Results of Fe precipitation from real leach solution varying the pH, temperature, solid/solution Na_2CO_3, H_2O_2 time and precipitation time. Tests were carried out with addition of 7vol% H_2O_2 to the leaching solution.

Test	pH	Temperature (°C)	Precipitation time (min)	H_2O_2 time (min)	Na_2CO_3 addition	Fe (%)	Al (%)	Li (%)
Fe.1	3.0	25	120	5	Solid	93.5 ± 0.6	47.7 ± 0.5	13.1 ± 0.2
Fe.2	3.0	80	120	5	Solid	96.0 ± 0.5	37.2 ± 0.8	3.0 ± 0.1
Fe.3	3.0	80	120	5	Solution	92.9 ± 0.5	35.5 ± 0.8	0.5 ± 0.1
Fe.4	3.0	80	120	120	Solution	97.4 ± 0.8	58.9 ± 1.6	8.7 ± 0.5
Fe.5	3.0	80	360	5	Solution	97.2 ± 1.2	45.6 ± 2.7	0.0 ± 0.4
Fe.6	2.0	80	120	5	Solution	93.2 ± 0.6	32.2 ± 0.8	0.1 ± 0.1

Table S5: Percentage of Fe, Li and Al extraction from real leach solution with PuroliteS950 in batch and continuous extractions.

Test	pH	Resin/solution (g/mL)	Flow rate (mL/min)	Extraction process	Fe (%)	Al (%)	Li (%)
IX.Fe.1	2.0	0,04	-	Batch	40.8 ± 0.3	17.8 ± 0.6	9.4 ± 0.2
IX.Fe.2	3.0	0,04	-	Batch	54.7 ± 0.3	27.4 ± 0.6	6.8 ± 0.4
IX.Fe.3	3.0	0,14	-	Batch	73.2 ± 0.4	34.0 ± 0.5	17.7 ± 0.3
IX.Fe.4	2.0	0,06	40	Continuous	30.0 ± 1.8	0.1 ± 2.3	0.01 ± 0.01
IX.Fe.5	2.0	0,14	40	Continuous	70.0 ± 1.3	0.5 ± 2.1	0.01 ± 0.01

Table S6: Results for Al precipitation from real leaching solution, Na_2CO_3 reagent type.

Test	pH	Temperature (°C)	Na_2CO_3 reagent	Al (%)	Li (%)
Al.1	5.0	80	Solid	71.1 ± 1.0	24.8 ± 0.3
Al.2	5.0	80	Solution	70.7 ± 0.9	24.2 ± 0.2
Al.3	5.0	25	Solid	78.5 ± 2.4	12.0 ± 0.6
Al.4	6.0	25	Solution	95.8 ± 1.9	6.3 ± 0.6

Table S7: Li precipitation percentages for tests with synthetic solution varying the initial Li concentration, at pH 10 and 80°C.

Test	Initial Li concentration(g/L)	Li precipitation(%)
Li.1	1.0	0 ± 0
Li.2	2.0	0 ± 0
Li.3	3.0	88.6 ± 1.2
Li.4	4.0	91.3 ± 1.6

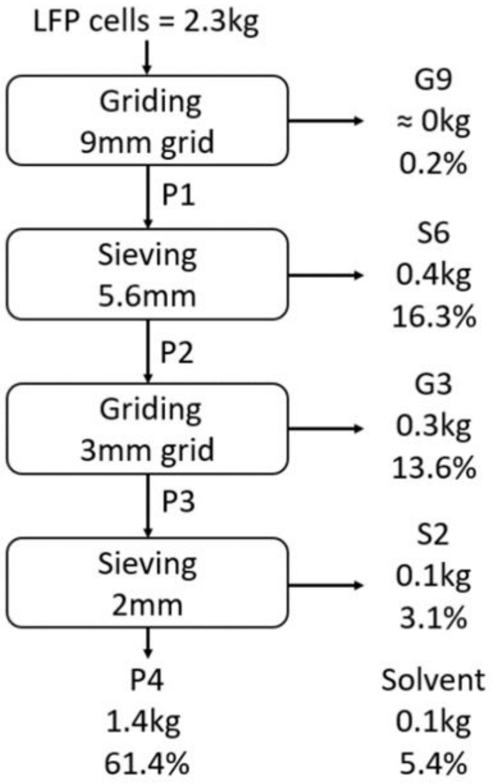

Figure S1: Flowchart of the mechanical steps used in comminution process of discharged LFP cells, indicating the total mass.

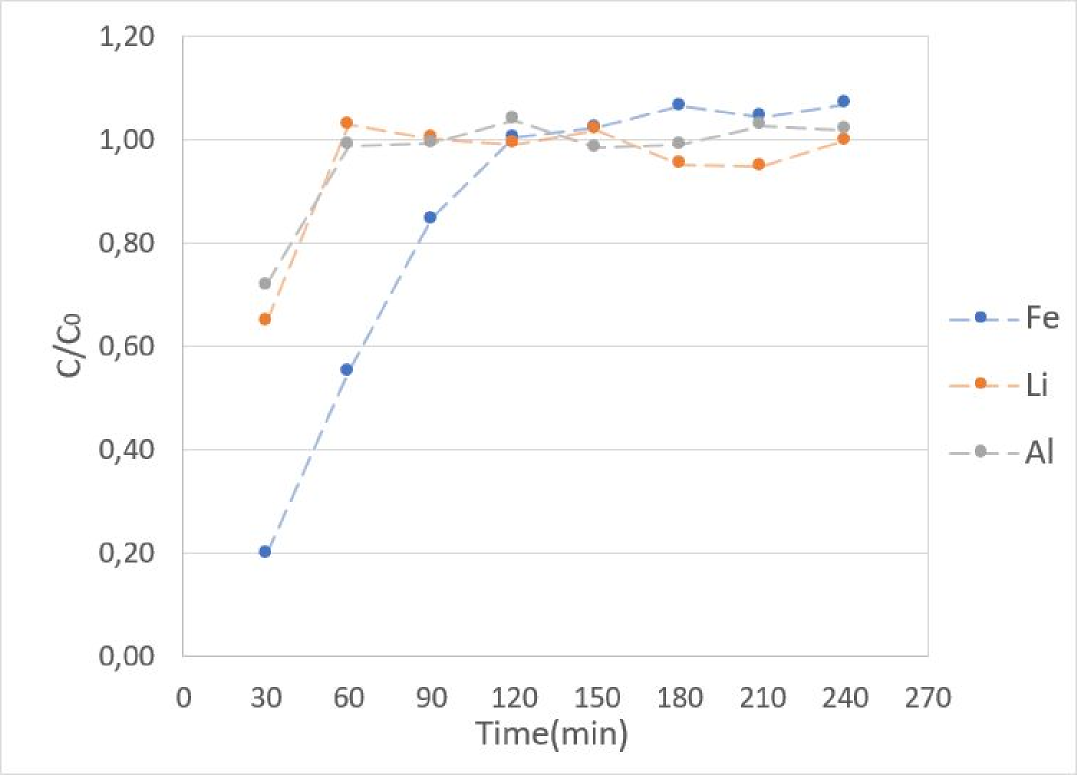

Figure S2: Breakthrough curve for Li, Fe and Al adsorption in PuroliteS950 continuous process. Conditions: 40mL/h flow rate, pH 2, T = 25°C and bed volume 10mL.

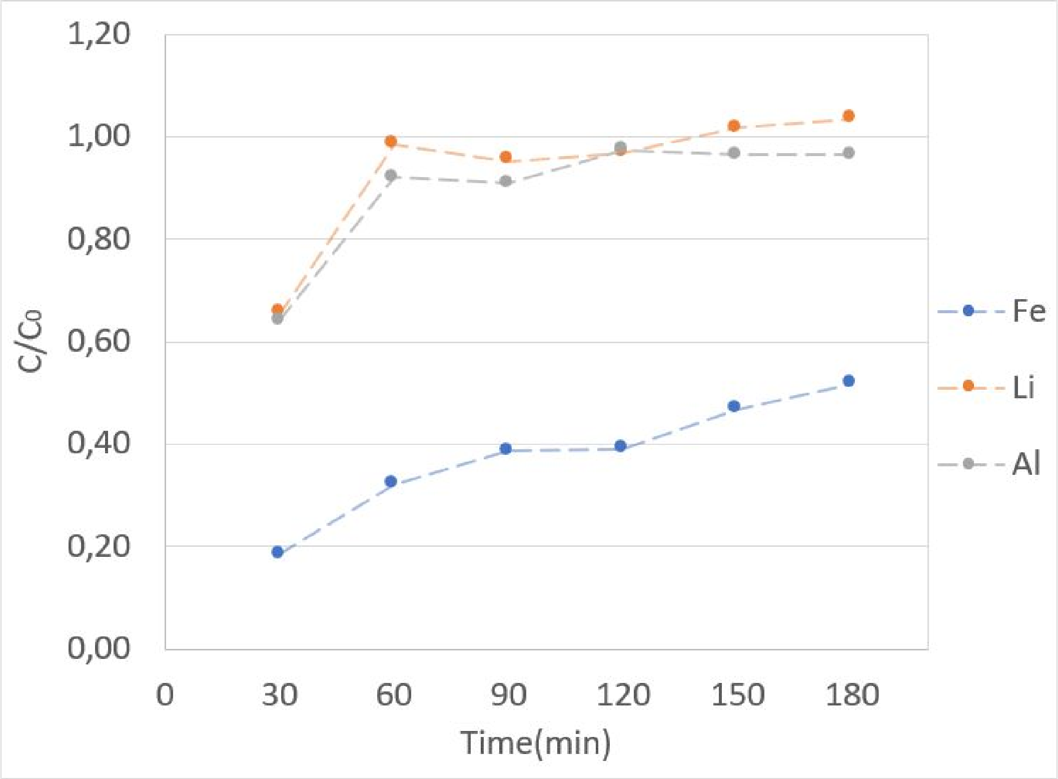

Figure S3: Breakthrough curve for Li, Fe and Al adsorption in PuroliteS950 continuous process. Conditions: 40mL/h flow rate, pH 2, T = 25°C and bed volume 20mL.

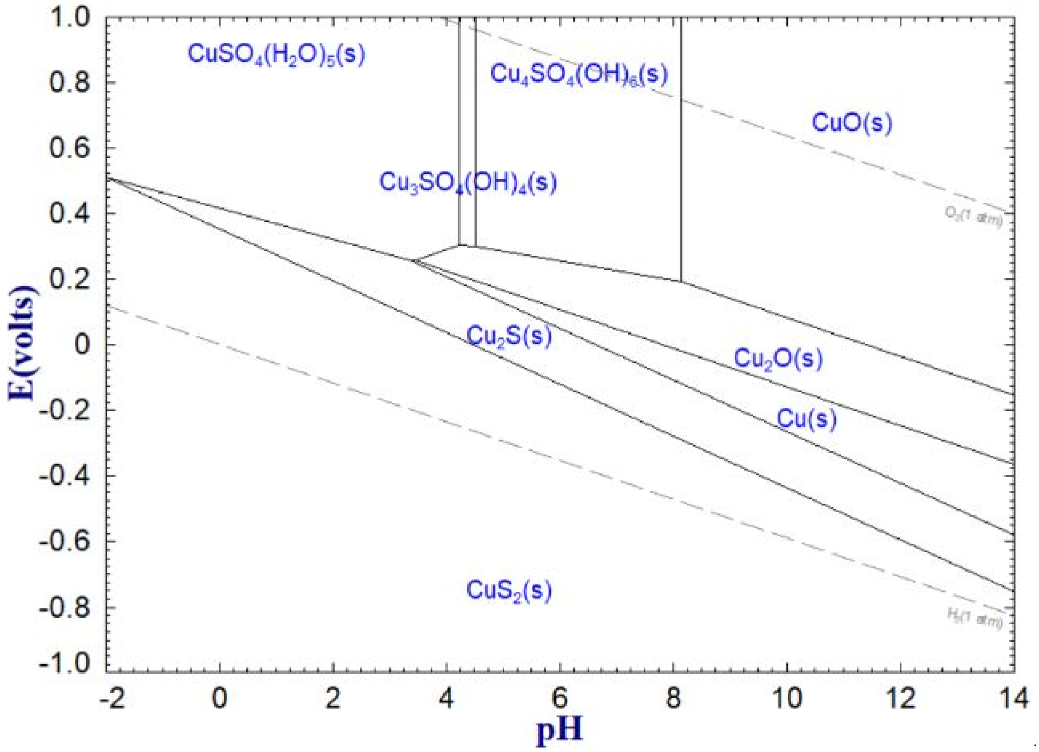

Figure S4: Pourbaix diagram of Cu-SO_4 system.

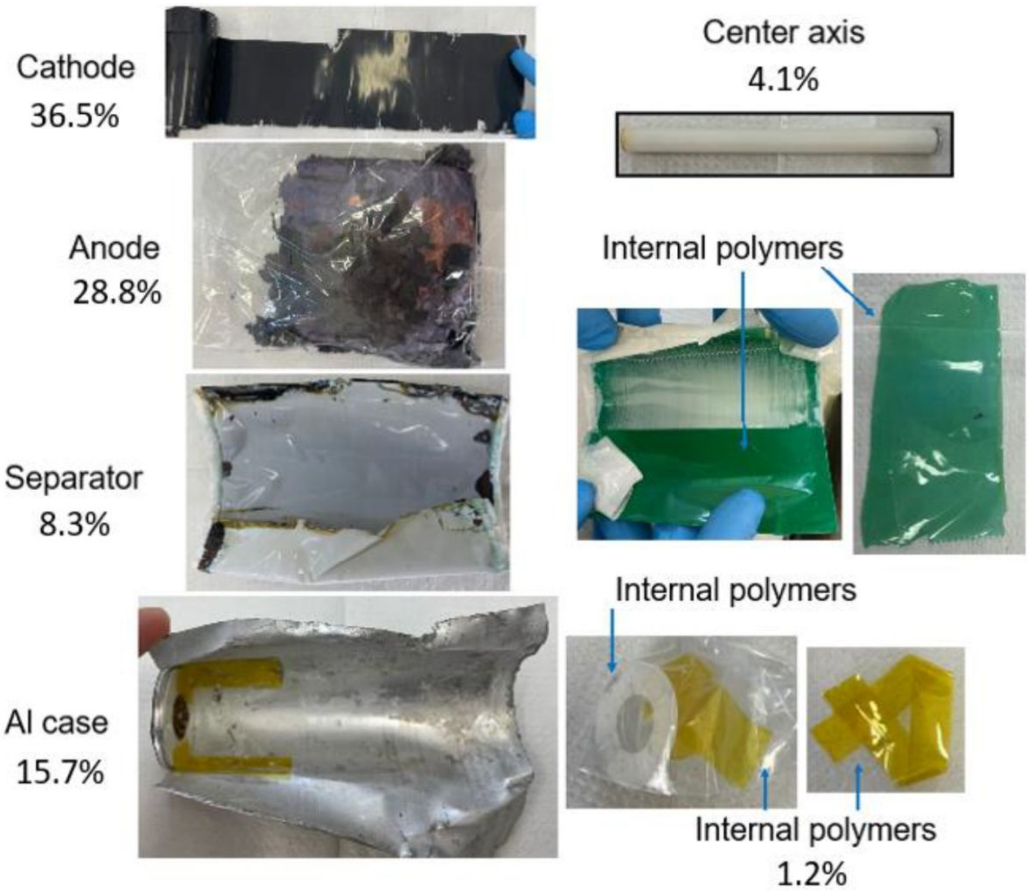

Figure S5: Dismantled LFP cell and mass percentages of each internal component of the cell.

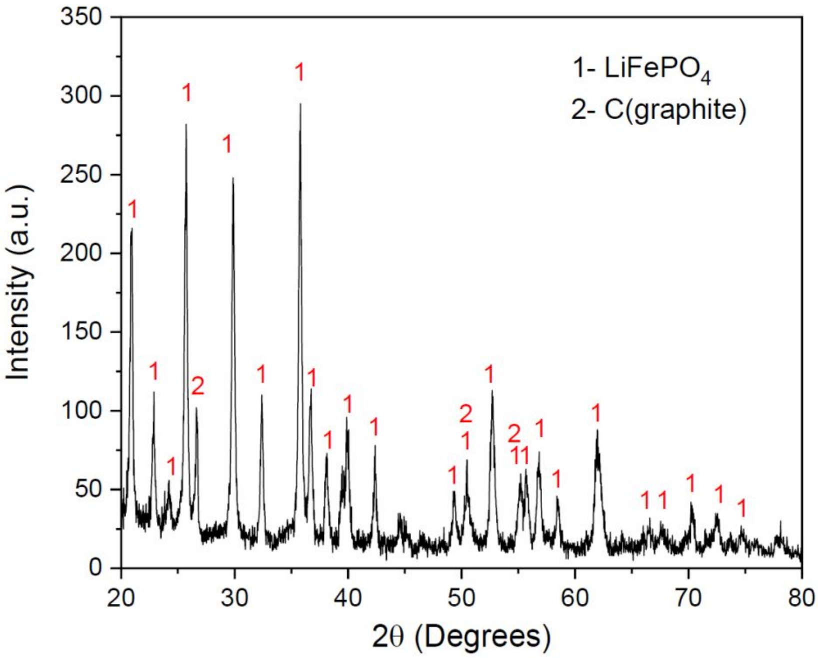

Figure S6: X-ray diffractogram of the cathode active material sample and the main phases detected.

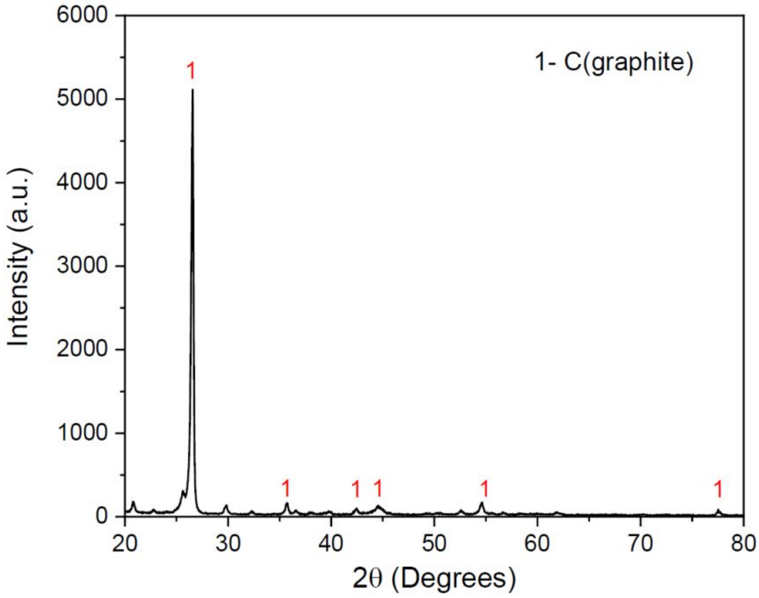

Figure S7: X-ray diffractogram of the anode active material sample and the main phases detected.

Table S8: Elements mass percentage in cells, obtained from determination of metals concentration in cathode and anode.

**Elements**	**Mass percentage (%)**	
	**Cathode**	**Anode**
Li	0.7 ± 0.05	0.1 ± 0.1
Fe	6.6 ± 0.2	-
Al	9.0 ± 0.7	-
Cu	-	14.9 ± 0.5
C	3.3 ± 0.6	11.6 ± 0.4

Table S9: Mass percentage of components in dismantled LFP cells.

**Cell components**			**Mass percentage (%)**
Electrodes	Anode	Graphite	13.8 ± 0.5
		Cu foil	15.0 ± 0.5
	Cathode	Active material	27.5 ± 0.7
		Al foil	9.0 ± 0.7
Plastics	Separator		8.3 ± 0.1
	Internal		1.2 ± 0.2
	Center axis		4.1 ± 0.2
Al case			15.7 ± 1.0
	Organic solvent		5.4 ± 0.6

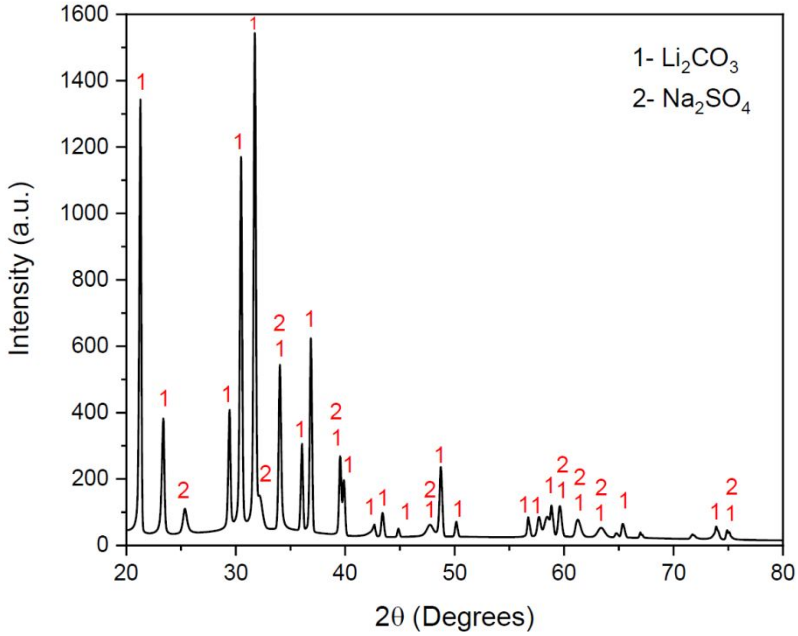

Figure S8: X-ray diffractogram of the Li_2CO_3 precipitate at pH 10.

Table S10:: Mass percentage of elements in $FePO_4$ and Li_2CO_3 products. Chemical analysis in ICP-OES.

**Elements**	**Mass percentage in $FePO_4$ (%)**	**Mass percentage in Li_2CO_3 (%)**
Li	0.2 ± 0.05	17.6 ± 0.1
Fe	33.1 ± 0.2	< 0.01
Al	2.4 ± 0.7	< 0.01
Cu	< 0.01	< 0.01
Na	1.1 ± 0.6	1.8 ± 0.3
